# Supplementary material for: Investigating the Role of Root Exudates in Recruiting Streptomyces Bacteria to the Arabidopsis thaliana Microbiome
Source: Front Mol Biosci. 2021 Jun 16;8:686110. doi: 10.3389/fmolb.2021.686110 (PMC8241931; doi:10.3389/fmolb.2021.686110)
Supplement: Supplementary file 1 [file DataSheet1.pdf]

Supplementary Material

1 Supplementary Figures and Tables

Table S1. See attached Excel spreadsheet.

Table S2. Primers and plasmids used in this study.

**Primers**			
**Name**	**Description**	**Sequence**	**Reference**
PRK341F	Universal primer pair that amplifies the V3-V4 region 16S rRNA gene.	5’-CCTACGGGAGGCAGCAG-3’	[1]
PRK806R		5’-GGACTACHVGGGTWTCTAAT-3’	
ermEKpnFwd	Used to amplify ermE* from the plasmid PIJ110257	CGA CTC GGT ACC AGC CCG ACC CGA GCA CGC	This study
ermENdeRev		CGA ACC CAT ATG GGG CCT CCT GTT CT	This study

Plasmids		
**Name**	**Description**	**Reference**
PIJ8660	Contains an optimised eGFP gene; aac^R.	[2]
PIJ10257	Used as a template from which to amplify ermEp*	[3]
PIJ8660/erm	Contains an optimised eGFP gene under the control of the constitutive ermE* promotor; aac^R.	This study

Table S3. Characteristics of F2 Levington Compost.

Parameter	Measurement	SAC Rating
pH	4.98	n/a
Phosphorous	880.5 mg/kg	Excessively high
Potassium	2508 mg/kg	Excessively high
Magnesium	6021mg/kg	Excessively high

Nitrate	5809.49 g/kg	n/a
Ammonium	192.18 g/kg	n/a
Organic matter	91.08 %	n/a

Table S4. Summary of stable isotope probing results displaying a list of genera that were identified as candidate autotrophs in either the rhizosphere or endosphere, as well as the genera (and their associated phyla) that were identified to be more than two-fold enriched in the heavy fractions (versus the light fractions) of samples from the rhizosphere or endosphere (or both, shown in red) suggesting they were metabolising labelled root exudates.

Autotrophic genera		Metabolisers of root exudates in the rhizosphere		Metabolisers of root exudates in the endosphere	
**Rhizosphere**	**Endosphere**	**Phylum**	**Genus**	**Phylum**	**Genus**
Bacteroides	*Amaricoccus*	Proteobacteria	*Ensifer*	Proteobacteria	*Sphingobium*
Bordetella	*Bacteroides*	Proteobacteria	*Sideroxydans*	Actinobacteria	*Jatrophihabitans*
Brevundimonas	*Bordetella*	Proteobacteria	*Pseudomonas*	Proteobacteria	*Novosphingobium*
Candidatus nitrotoga	*Brevundimonas*	Proteobacteria	*Sinorhizobium*	Proteobacteria	*Methyloferula*
Candidimonas	*Candidatus nitrotoga*	Proteobacteria	*Telluria*	Proteobacteria	*Sinorhizobium*
Delftia	*Frateuria*	Proteobacteria	*Herbaspirillum*	Proteobacteria	*Shinella*
Frateuria	*Geobacter*	Proteobacteria	*Shinella*	Proteobacteria	*Pseudomonas*
Hirschia	*Heliobacterium*	Proteobacteria	*Limnobacter*	Proteobacteria	*Acidovorax*
Methylibium	*Hirschia*	Proteobacteria	*Massilia*	Proteobacteria	*Ensifer*
Methylobacterium	*Longilinea*	Proteobacteria	*Azohydromonas*	Proteobacteria	*Rhizobium*
Nitrosospira	*Luteimonas*	Proteobacteria	*Sphingopyxis*	Proteobacteria	*Agrobacterium*
Paracoccus	*Magnetospirillum*	Proteobacteria	*Chelatococcus*	Planctomycetes	*Blastopirellula*
Prochlorococcus	*Methylibium*	Proteobacteria	*Aquincola*	Proteobacteria	*Azospirillum*
Sterolibacterium	*Methylobacterium*	Chloroflexi	*Levilinea*	Proteobacteria	*Sphingopyxis*
Woodsholea	*Methyloversatilis*	Proteobacteria	*Altererythrobacter*	Proteobacteria	*Roseomonas*
	Nannocystis	Proteobacteria	*Hyphomicrobium*	Proteobacteria	*Lacibacterium*
	Paracoccus	Proteobacteria	*Methylocella*	Proteobacteria	*Pseudoxanthomonas*
	Prosthecomicrobium	Proteobacteria	*Pseudoxanthomonas*	Proteobacteria	*Rhodanobacter*
	Rhodocista	Cyanobacteria	*Chroococcidiopsis*	Planctomycetes	*Pirellula*
	Thermoleophilum	Proteobacteria	*Rhizobium*	Proteobacteria	*Telluria*
	Woodsholea	Proteobacteria	*Duganella*	Proteobacteria	*Massilia*
		Proteobacteria	*Asticcacaulis*	Proteobacteria	*Thermomonas*
		Proteobacteria	*Solimonas*	Chloroflexi	*Chloroflexus*
		Proteobacteria	*Thermomonas*	Firmicutes	*Chelativorans*
		Proteobacteria	*Acidovorax*	Planctomycetes	*Gemmata*
		Planctomycetes	*Pirellula*	Proteobacteria	*Aquincola*
		Proteobacteria	*Aetherobacter*	Proteobacteria	*Limimonas*
		Firmicutes	*Pelotomaculum*		

Table S5. Buffer and media recipes [see also 4]

Media	Component	g L^{-1} dH_2O
Soya Flour Mannitol (SFM) agar	Soy flour	20
	Mannitol	20
	Agar	20
Maltose-Yeast extract-Malt extract (MYM) agar	Maltose	4
	Yeast Extract	4
	Malt Extract	10
	Agar	18
ISP2 Agar	Yeast Extract	4
	Maltose	10
	D-glucose	4
	Agar	20
2xYT	Bacto-tryptone	16
	Yeast extract	10

	NaCl	5
Silwett L-77 amended Phosphate Buffered Saline (PBS-S)	$NaH_2PO_4.H_2O$	6.33 g
	$Na_2HPO_4.H_2O$	16.5 g
	Silwet L-77	200 µl added after autoclaving
Minimal agarose media	NH_4SO_4	1
	KH_2PO_4	0.5
	$MgSO_4.7H_2O$	0.2
	$FeSO_4.7H_2O$	0.01
	Agarose	6
	Trace element solution	2 ml added after autoclaving
	**Optional carbon source:**	
	Mannitol **or**	5
	Maltose **or**	5

	Sucrose **or**<br>Glucose **or**<br>SA **or**<br>Sodium citrate	5<br>10<br>0.069<br>1
Trace element solution	$ZnCl_2$<br>$FeCl_3.6H_2O$<br>$CuCl_2.2H_2O$<br>$MnCl_2.4H_2O$<br>$Na_2B_4O_7.10H_2O$<br>$(NH_4)_6Mo_7O_{24}.4H_2O$	0.04<br>0.2<br>0.01<br>0.01<br>0.01<br>0.01
Murashige and Skoog (MSk) Agar	Murashige and Skoog salts (Duchefa Biochemie, Harlem, Netherlands)<br>Sucrose<br>Agar	4.43<br>10<br>8

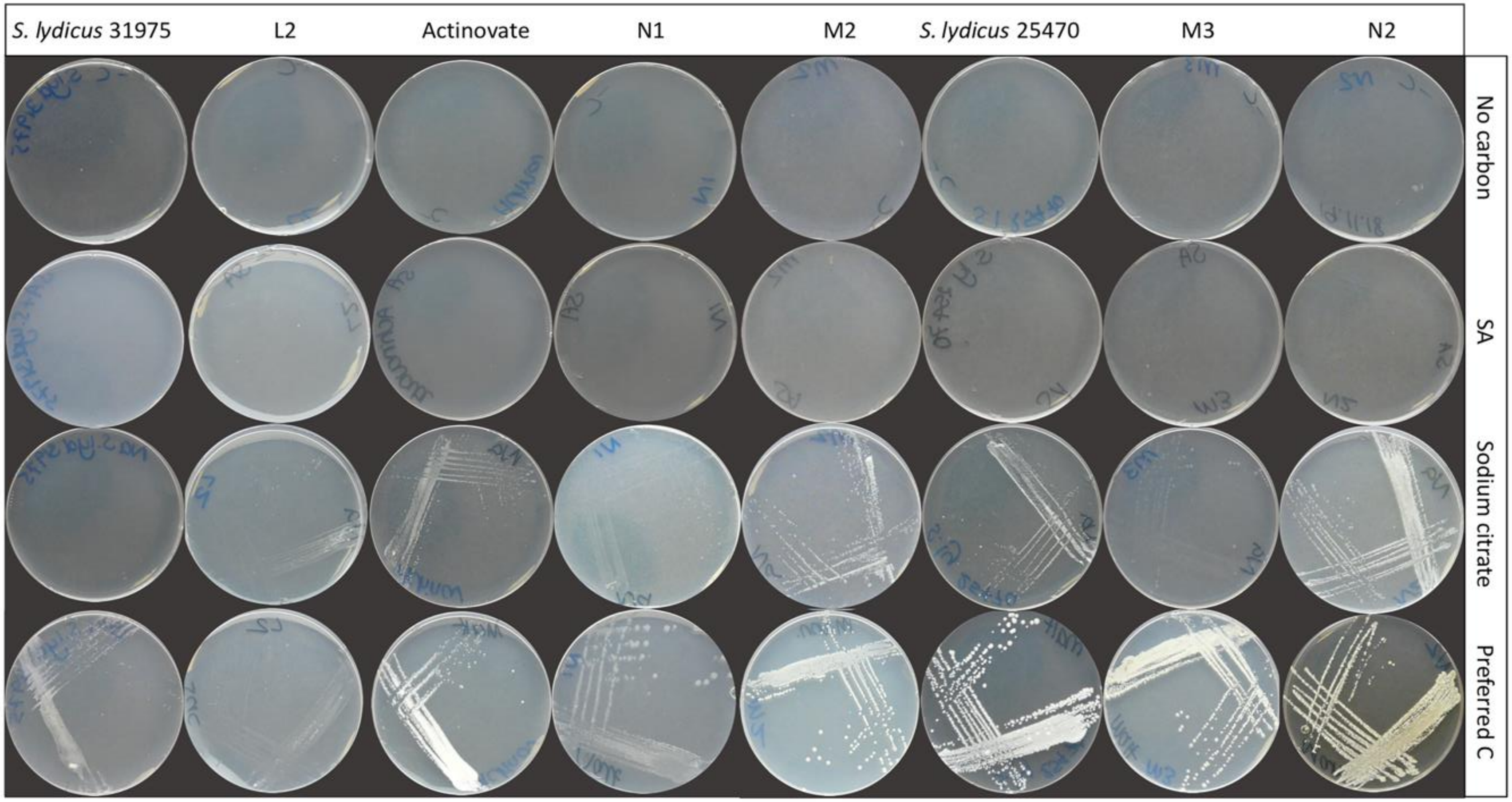

Figure S1. *Streptomyces* isolates (as indicated) grown on minimal medium agarose plates containing either no carbon, 0.5 mM salicylic acid (SA), 3.875 mM sodium citrate or their preferred carbon source.

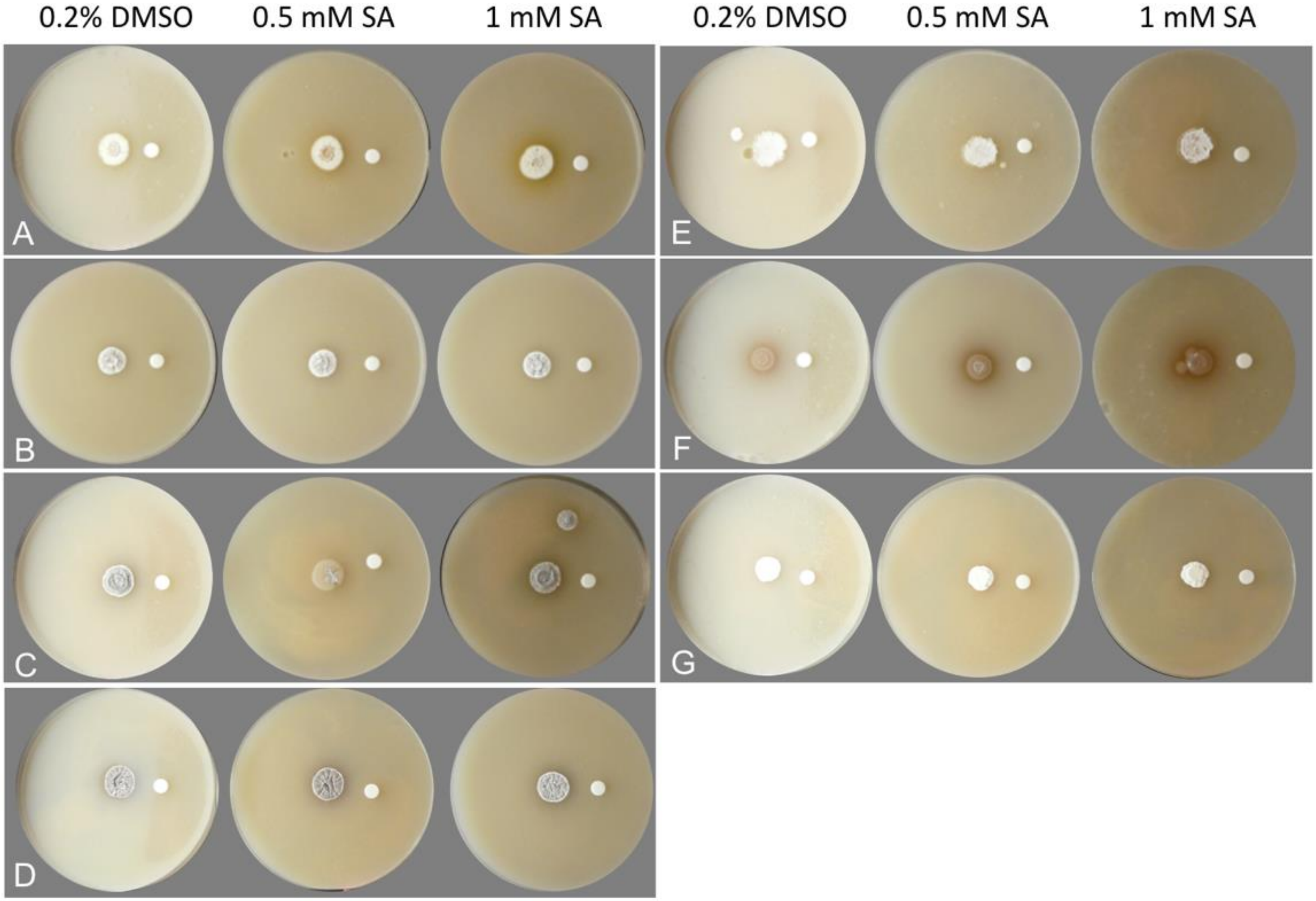

Figure S2. Salicylic acid chemoattractant assays. Streptomycete colonies were grown next to disks soaked in 0.1% DMSO (as a control for the SA solvent), 0.5 mM SA or 1 mM SA. Images were taken after 10 days of growth at 30°C. All assays were carried out on SFM agar media (Table S5). *Streptomyces* strains **A)** N2, **B)** N1, **C)** M3, **D)** *Streptomyces coelicolor* M145, **E)** M2, **F)** L2 and **G)** Actinovate were tested for their response to SA.

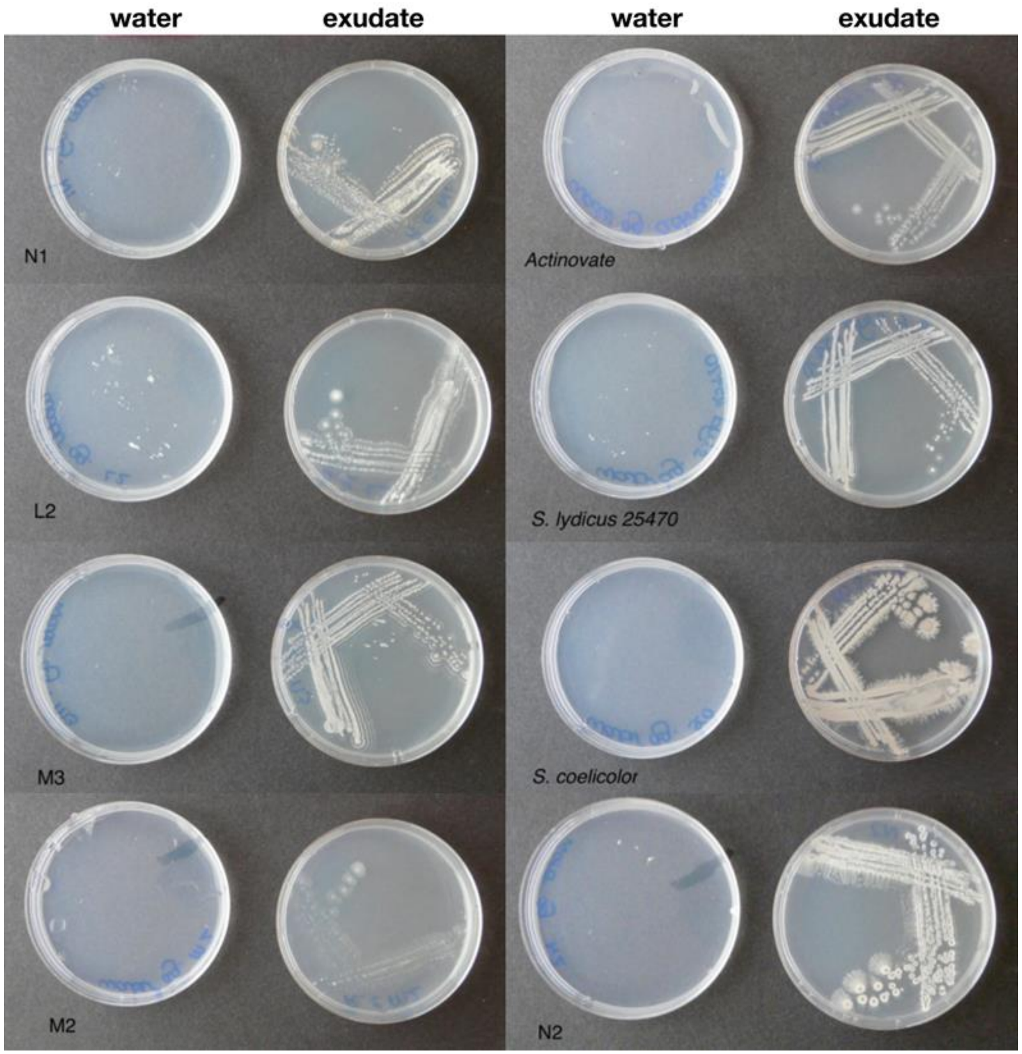

Figure S3. *Streptomyces* isolates growing on root exudates as a sole food source. Strains N1, L2, M3, M2 and N2 were isolated from surface washed *A. thaliana* roots. *Actinovate* is the *S. lydicus* WYEC106 cultured from the horticultural product Actinovate. *S. lydicus* 25470 was acquired from the American Type Culture Collection (see Table S1 in Additional File 1 for more details).

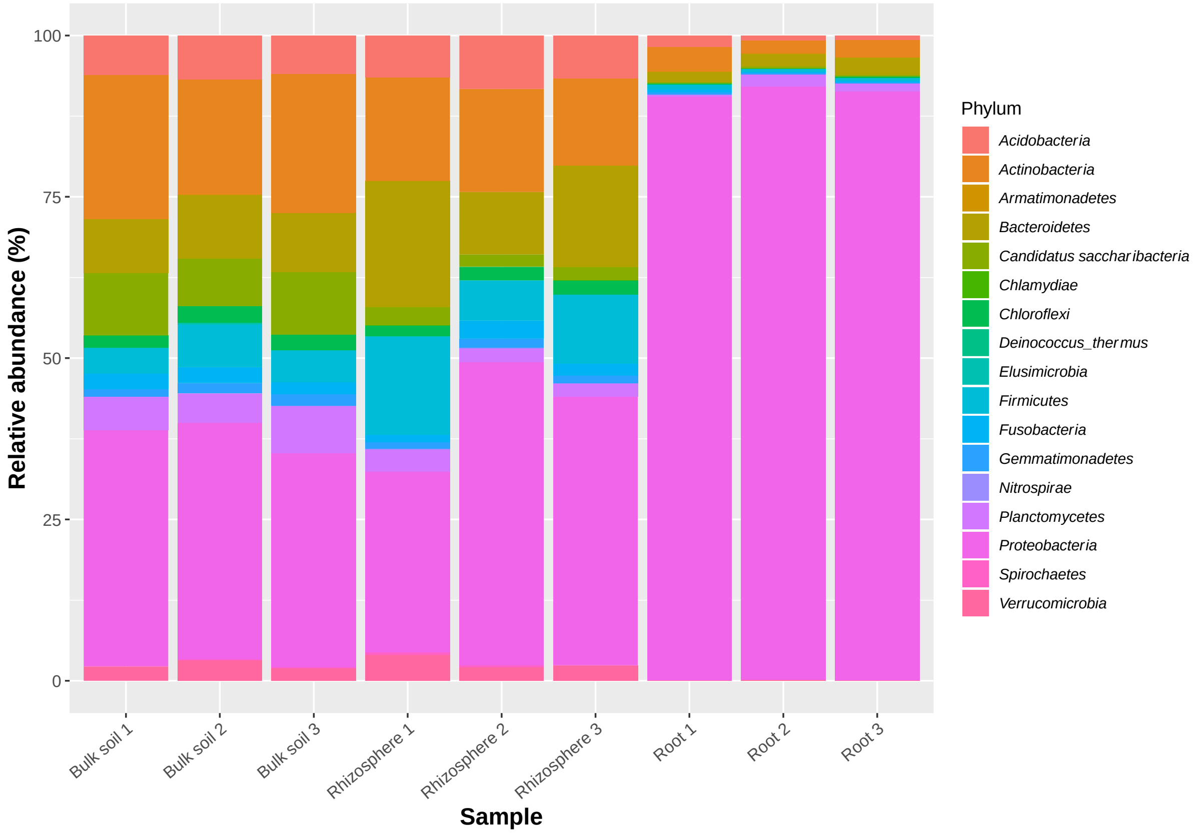

Figure S4. The relative abundance (%) of bacterial phyla in samples from the bulk soil, rhizosphere and root compartments of *Arabidopsis thaliana* grown in compost (n=3 replicate samples per compartment).

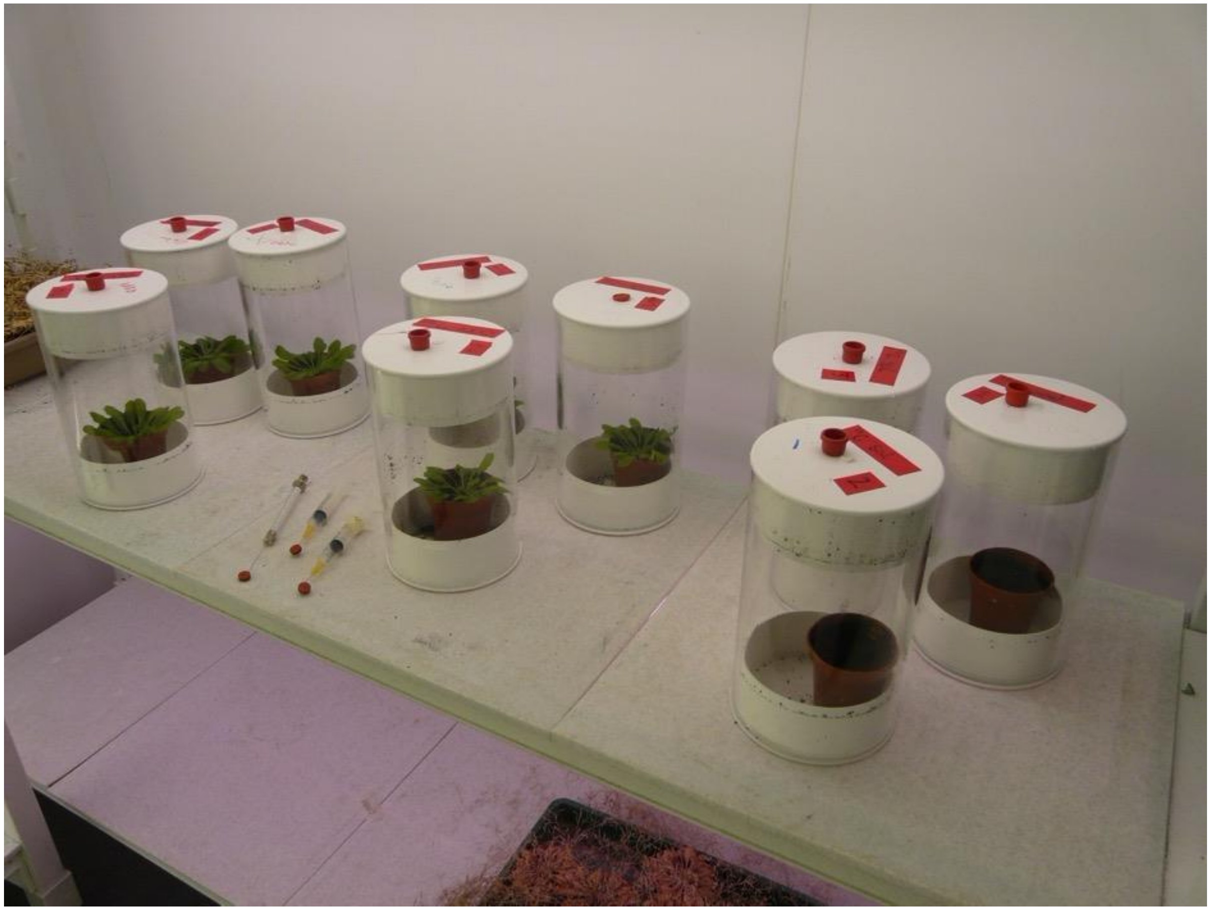

Figure S5. ^{13}C DNA stable isotope probing with *A. thaliana* plants.

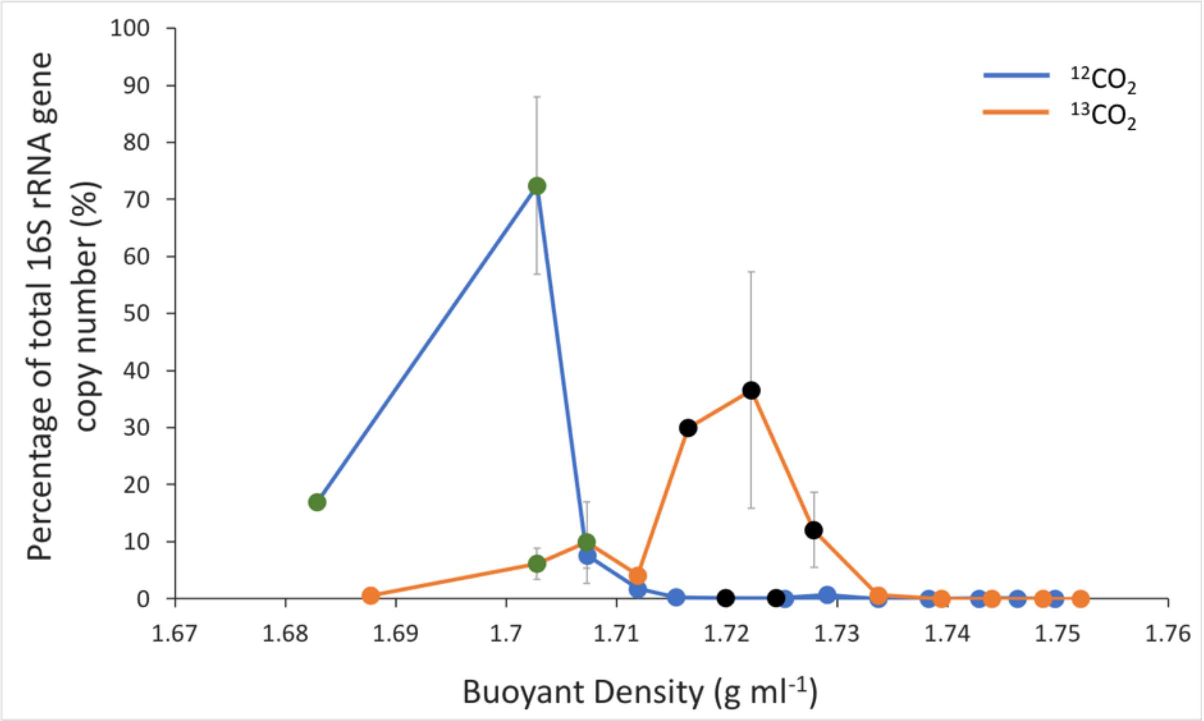

Figure S6. 16S rRNA gene copy number across buoyant density fractions of DNA isolated from root samples of *A. thaliana plants*incubated under either $^{12}CO_2$ (blue) or $^{13}CO_2$ (orange) conditions. Copy number is shown as a percentage of total copy number per sample. N=2 replicate measurements on the same fraction. Bars represent standard errors. Green and black dots represent the “light” and “heavy” fractions, respectively, that were pooled in each treatment and submitted for 16S rRNA gene amplicon sequencing.

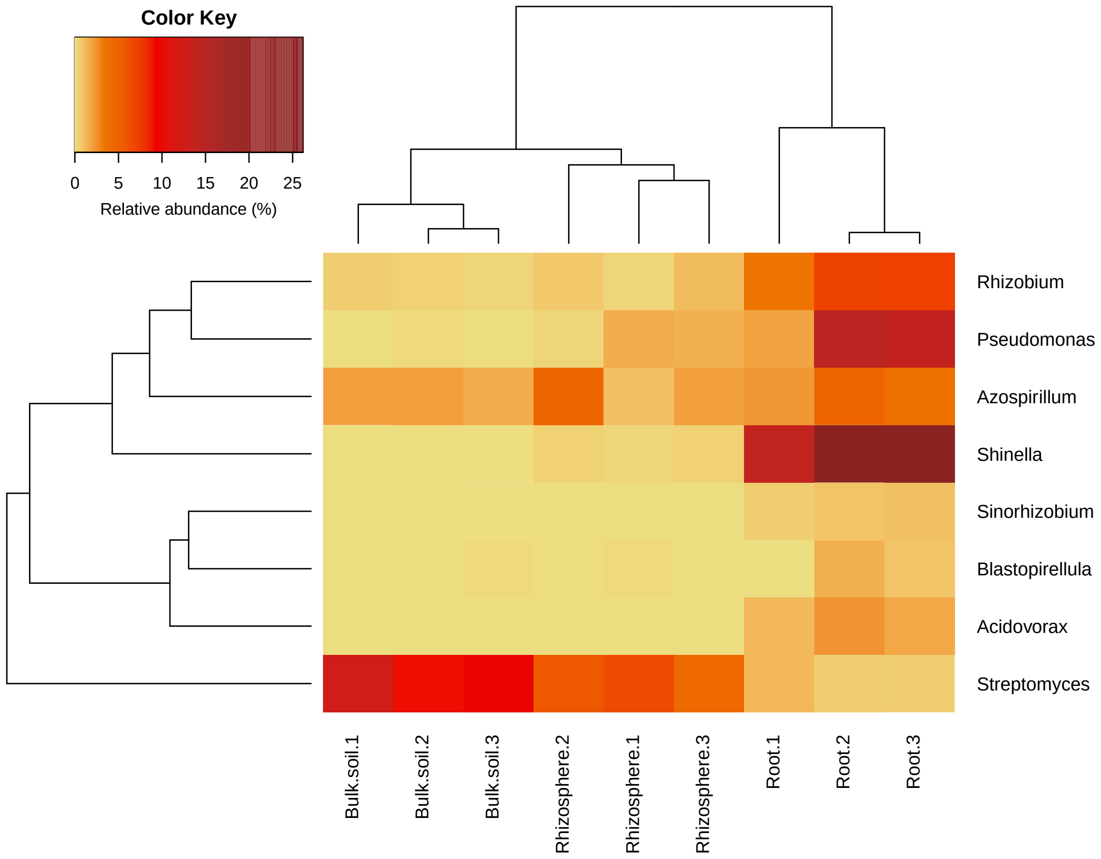

Figure S7. The abundance of the eight bacterial genera metabolising the greatest quantities of labelled exudates in the endophytic compartment of *A. thaliana*(determined by ratios of abundance in 13CH to 12CH fractions). Their relative abundances (%) are shown across the bulk soil, rhizosphere and root compartments (light yellow to brown indicates increasing relative abundance). N = 3 replicate plants in individual pots. *Streptomyces* species were not metabolising root exudates, nor were they enriched in the endophytic compartment, but are shown for comparison. Clustering represents Bray-Curtis dissimilarities.

References

1. Yu, Y., et al., *Group-specific primer and probe sets to detect methanogenic communities using quantitative real-time polymerase chain reaction.* Biotechnol Bioeng, 2005. **89**(6): p. 670-9.
2. Sun, J., et al., *Green fluorescent protein as a reporter for spatial and temporal gene expression in Streptomyces coelicolor A3(2).* Microbiology, 1999. **145 (Pt 9)**: p. 2221-7.
3. Hong, H.J., et al., *The role of the novel Fem protein VanK in vancomycin resistance in Streptomyces coelicolor.* J Biol Chem, 2005. **280**(13): p. 13055-61.
4. ActinoBase. *ActinoBase: an Actinomycete Community Wiki*. Available from: www.actinobase.org.
